# Introducing a global database of entomopathogenic fungi and their host associations

**DOI:** 10.1038/s41597-024-04103-4

**Published:** 2024-12-21

**Authors:** Frederik C. De Wint, Soun Nicholson, Qian Qun Koid, Shafia Zahra, Georgia Chestney-Claassen, Jaya Seelan Sathiya Seelan, Jie Xie, Shuang Xing, Tom M. Fayle, Danny Haelewaters

**Affiliations:** 1 Biology Centre of the Czech Academy of Sciences, Institute of Entomology, Department of Ecology, Ceske Budejovice, Czech Republic; 2 Department of Zoology, Faculty of Science, University of South Bohemia, Ceske Budejovice, Czech Republic; 3 Research Group Mycology, Department of Biology, Ghent University, Ghent, Belgium; 4 Royal Botanic Gardens, Kew, Richmond, United Kingdom; 5 Institute for Tropical Biology and Conservation, Universiti Malaysia Sabah, Kota Kinabalu, Sabah Malaysia; 6 School of Ecology, Shenzhen Campus of Sun Yat-sen University, Shenzhen, Guangdong 518107 China; 7 School of Biological and Behavioural Sciences, Queen Mary University of London, London, United Kingdom

**Keywords:** Ecological epidemiology, Zoology

## Abstract

Pathogens significantly influence natural and agricultural ecosystems, playing a crucial role in the regulation of species populations and maintaining biodiversity. Entomopathogenic fungi (EF), particularly within the Hypocreales order, exemplify understudied pathogens that infect insects and other arthropods globally. Despite their ecological importance, comprehensive data on EF host specificity and geographical distribution are lacking. To address this, we present EntomoFun 1.0, an open-access database centralizing global records of EF–insect associations in Hypocreales. This database includes 1,791 records detailing EF species, insect host taxa, countries of occurrence, life stages of hosts, and information sources. EntomoFun 1.0 is constructed based on 600 literature sources, as well as herbarium specimens of the Royal Botanical Gardens, Kew. This database is intended to test hypotheses, identify knowledge gaps, and stimulate future research. Contents of the EntomoFun 1.0 database are visualized with a global map, taxonomic chart, bipartite community network, and graphs.

## Background & Summary

Pathogens are key players in natural and agricultural ecosystems. This has become even more apparent in times of climate change and anthropogenic introductions^1^. Pathogens hold ecological significance as regulators of species populations and communities^2,3^. Host–pathogen dynamics are inherently tied to the density of host populations^4–6^. As a result, pathogens play a crucial role in preserving biodiversity and primary production by controlling populations of susceptible species^1^. Studying the impacts of pathogens in ecosystems is critical for conservation management in order to maintain ecological stability and resilience^2^. Herein, the ability to recognize the hosts of pathogens is of utmost significance; it forms the basis for effective disease management and ecological conservation^2,7^. However, there is a lack of knowledge on species associations and host specificity (especially at lower taxonomic levels), which complicates comprehension of understudied pathogens^8^.

A prime example of understudied pathogens are entomopathogenic fungi (EF), which infect and kill insects and other arthropods of many different lineages across the globe^9,10^. Infection by EF is mediated by spores that attach to the host and penetrate through the cuticle^11^. Via hyphal growth, the fungus then builds up mycelium inside the hemocoel. If the temperature and humidity are favorable, the fungus then emerges from the host body. Often fruiting bodies are formed that finally produce new spores. An infamous example of EF is the “zombie–ant fungus” most commonly referred to as *Ophiocordyceps unilateralis* sensu lato (s.l.) in the literature. Ants and other insects infected by *Ophiocordyceps* fungi are manipulated to relocate themselves according to fungal preference before they die^12,13^. The resulting position provides the fungus with adequate ambient humidity for its growth and optimizes spore dispersal. Insects and other arthropods have a global biomass similar to that of humans and their livestock combined^14^, and their species diversity is estimated to be 5.5 and 1.5 million worldwide, respectively^15^. Hence, EF infecting them are likely to be a key group in global ecosystems.

However, despite the high number of insect species, little is known about the ecological impact of EF on this widespread group, especially in natural contexts. One of the main limitations in studying EF is the lack of integrative and centralized information for host specificity and geographical distribution^16^. While some EF are generalists in nature, meaning they are capable of infecting a wide range of hosts, others are more specialized and often restricted to a single host species (and for a number of EF species even to a single life stage of that host)^3,10^. The exploration of species records along a spatial scale indicates how widespread or endemic they may be. Knowledge on host and geographical ranges allows predictions about which species are candidates to become invasive and where invasions could occur^17^, as well as which species might face extinction and where those extinctions might occur^18,19^. This information is crucial in light of ongoing climate change, habitat modification, and introduction of non-native taxa including biological control agents^20,21^.

Progress in ecological research on EF has been hampered due to a number of limitations. To date, the extent of the host range of many EF species has not been thoroughly evaluated. Many papers present partial, incomplete, or assumed host ranges^9,22^. Second, many descriptions and reports on these fungal species present limited information on their hosts. This pertains to their taxonomy – often only higher taxonomic-level identifications are provided – and life stages – it is not always clear which life stage is infected. This is because infected insects are generally tiny, with a quickly decaying cuticle often fully covered with fungal mycelium. This means that identification of the insect is often difficult, especially in the field. Finally, EF have been extensively investigated in some regions, but remain unstudied in others. This pattern is also seen in other groups of understudied fungi^23,24^. Much of the available host and distributional information of EF is scattered in the literature and herbaria. Thus, these data are currently inaccessible as a whole.

Here we present EntomoFun 1.0, an open-access database that centralizes information on EF–insect associations and their distributions, with focus on the hyperdiverse fungal order Hypocreales (phylum Ascomycota, class Sordariomycetes). Our database provides a global summary of observation records, including information on EF species, insect host taxon, country of occurrence, host life stage, and primary source reference. The database is constructed through careful assimilation of observations available in the literature, supplemented with herbarium specimen records. Araújo and Hughes^9^ presented data on all known EF species and the insect orders they infect. Therefore, the Hypocreales species list provided in their work was used as a baseline for the construction of EntomoFun 1.0. This database is innovative as it comprises individual data records, focusing on lower taxonomic identities, occurrences, and host life stages. Most records in EntomoFun 1.0 belong to the fungal genera *Cordyceps* and *Ophiocordyceps*, which represent relatively well-explored and remarkably diverse taxa of EF that are known to induce “zombie” host behavior^9^.

Providing EntomoFun 1.0 as open data provides numerous advantages, including reproducibility and transparency^25,26^. As the data can be used to address multiple research questions, making our database freely accessible removes the need for other scientists to go through the process of assimilating, cleaning, and curating data themselves. EntomoFun 1.0 is in accordance with the FAIR principles^27^. Different formats of the database are provided to facilitate direct usage, including summary tables to concretize EF–host network interactions as well as an EF species list including basionyms^28^ and synonyms to demonstrate taxonomic changes over time.

The database is stored in Dryad^29^ and can be accessed through the following link: 10.5061/dryad.1zcrjdg17. We aim to publish future versions of EntomoFun to include new records from the field and data from other sources, such as citizen science repositories (e.g., iNaturalist, https://www.inaturalist.org/) and MyCoPortal (https://www.mycoportal.org/portal/). In future versions, we also aim to include records of EF from groups other than Hypocreales.

With this database we aim to (1) present an openly accessible data repository with currently known insect hosts and occurrence data for each EF species in Hypocreales, (2) provide these data formatted for analyses to address contemporary questions in EF macroecology, (3) identify knowledge gaps in host range and geographical distribution of EF species, and (4) stimulate future research venues and field studies.

## Methods

### Creation of the database

The EntomoFun 1.0 database was constructed with the aim to include all known EF–host associations, only including fungi in Hypocreales and their insect hosts. Note that the recently erected family Polycephalomycetaceae^30^ was not considered here. Primary and secondary journal publications as well as other reliable sources were thoroughly searched for observations of EF species and any host insect species that they infect. We defined “primary literature” as those works that present original observations included in the database, and “secondary literature” as those works that provide summaries of observations from primary literature. Associations were recorded along with their metadata including: country of occurrence, host life stage, and primary source reference. Data collation was done in 2021–2023. Only records found in the field were included (e.g., primary forests, disturbed habitats, botanical gardens, and plantations); artificial records from lab or field tests were excluded.

Araújo and Hughes^9^ presented a list of all sexual morphs of EF species from the order Hypocreales known to science at the time, along with the insect host orders they infect. This list was used as a baseline for our database collation. In mycology, multiple asexual and sexual stages have been historically assigned to different taxonomic lineages. The adoption of the One Species One Name principle in 2011 resulted in numerous sexual–asexual connections and taxonomic consequences^31,32^. In our database, we provided information only for the sexual stage^9^. We added EF species newly described since Araújo and Hughes^9^, for which the sexual morph is known. The resulting species list was expanded to include the currently accepted name, basionym, and synonyms through the use of MycoBank^33^ (file_six). We excluded EF species that infect Hemiptera specifically. This concerns a hyperdiverse group of more than 180 species^9^ and will be addressed in a future version of EntomoFun.

The main body of our database was obtained through an exhaustive literature search, by means of a collation protocol. These literature-based data were supplemented with information from specimens deposited in the herbarium of the Royal Botanical Gardens, Kew. Label information was examined and transcribed directly from herbarium specimens. Criteria for herbarium specimen information were identical to those of literature observations described below.

The literature search protocol involved the use of a search engine. Since we were looking for any legitimate records of host species infected by EF, we chose a rather broad approach for our literature search. Thus Google Scholar was used as the core for data collation. This search engine was chosen over other search engines such as Web of Science or Scopus, which employ narrower filters that result in a more limited coverage of literature globally (e.g., lower coverage of non-English language and non-journal publications). Non-English literature was translated either by proficient speakers of the language or using Google Translate when native speakers were unavailable, and then evaluated with care by the authors.

For each EF species, searches were done following a general protocol outlined below, focusing on one name at a time (one of: currently accepted name, basionym, or synonyms). A format of “Genus species-epithet” was employed to retrieve as much relevant literature as possible (e.g., “*Ophiocordyceps myrmecophila*” without the quotation marks). Observations were recorded in the database as single rows, providing information on these data fields: the observed EF species, host insect taxon (to the lowest taxonomic level reported), country of occurrence, host life stage, and reference to primary literature. New combinations of these fields were added to the database as new rows, such that each record was unique. In case of identical observations from different sources (i.e., same fungal and arthropod species and life stage in the same country), the multiple sources were added to the same row. Hence our database can be thought of as presenting a single row for each arthropod–fungus taxonomic combination per life stage and per country. During the search using a given EF species name, observations of any of the other EF species from publications found were recorded as well. To ensure maximum coverage, we considered both the primary and secondary literature. The screening of literature for a given EF species name was halted when 20 consecutive results from the search engine did not yield any new or previously recorded observations for the database.

As EF taxonomy has been through many changes, it was important to keep track of original species descriptions. Hence we also included the originally reported taxon name of EF for each individual record (based on the primary literature from which the information originated). This allows keeping track of authentic observations in case of future taxonomic revisions. As the secondary literature does not always present observations in their original format (e.g., updated names according to taxonomic revision, or assumptions made on the observation), we verified the information in these sources by checking the primary literature. On rare occasions, the primary literature was inaccessible (e.g., in the case of old literature) and the most likely reported name was determined using the publication year in combination with Index Fungorum^34^ and additional literature.

We adopted a conservative approach in our database. This means that we only included record information at a given taxonomic level in the database when we were completely certain of the identity at that level. For example, when “bee” was mentioned as a host, this was not conclusive enough to indicate a lower taxonomic level than the superfamily Apoidea^35^. Similarly, “wasp” was recorded as the suborder Apocrita, since no distinction can be made between sting-bearing wasps (in Aculeata) and parasitoid lineages^35^.

New records to the database were abundantly available during the early stages of data compilation, and gradually became scarcer over the course of data collation. Although multiple papers from the literature search seemed to be irrelevant at first sight, several such papers unexpectedly provided new records. Older primary sources of significant relevance were often cited by the secondary literature but unavailable through Google Scholar. They were accessed through institutional libraries and inter-library loans and subsequently searched for additional records. Often, these sources were without text recognition and thus could not be computer-searched. No records were found for some species because of records in old and inaccessible literature. In these cases, Index Fungorum^34^ was consulted to obtain at least the information on the host and locality for the type of the respective EF species.

### Cleaning and editing of the database

The recorded taxon names of EF and insects were checked for spelling mistakes, and additional columns were created with current nomenclature. Misspelled and outdated taxon names were identified using the R package *taxize*^36^, which allows comparison of taxa with taxonomic reference databases, Index Fungorum^34^ for EF and the GBIF Backbone Taxonomy^37^ for insect hosts. Low alignment scores between our data and these databases indicated taxon names in need of correction. This correction was done manually, by changing respective taxon names according to current nomenclature presented by the same two reference databases. In rare cases where GBIF was incomplete or insufficient to determine the current host taxon, we consulted the Taxonomy Browser of the National Center for Biotechnology Information (NCBI)^38^ and additional relevant taxonomic literature (e.g., for species of Hepialidae in the order Lepidoptera).

Additional attention was paid to cases where EF species have a complex taxonomic history. Throughout the literature, the same records were sometimes identified differently by various sources. This was due to misidentifications, confusing nomenclature, and taxonomic novelties. For such difficult taxonomic cases, we took notes and used them along with the respective literature and Index Fungorum^34^ to obtain the currently accepted name. When taxonomic accuracy of a record was less certain, we included the respective potential identity through means of “cf.” (“confer”, translates from Latin to “near”) or the slash symbol “/” (when listing the possible identities).

Following the many historical changes in EF taxonomy, several species were shown to be synonyms. However, due to lack of agreement by the mycological community on which names to use, these were retained as separate species in the major phylogenetic reorganization of Sung and colleagues^39^, where these species were placed in newly proposed higher taxa. An example is *Ophiocordyceps gentilis*, which has been inconsistently synonymized with two species throughout the literature, i.e., *Cordyceps oxycephala*^40,41^ and *C. sphecocephala*^42,43^. The latter is more commonly accepted. While the name *O. gentilis* refers to a synonym of another species, Sung *et al*.^39^ revised *Cordyceps gentilis* to *Ophiocordyceps gentilis*, changing the genus but retaining its status as a separate species. Such complex taxonomy issues deserve specific attention to be resolved, in the case of *O. gentilis* with detailed study of the limited number of specimens available in fungaria. Since verification of species status is currently beyond the scope of this work, we adopted the approach of Sung and colleagues^39^ and retained such cases as separate species in our database.

Higher-level taxa of both EF and insect species were also included in our database. Taxonomic levels of EF were based on Index Fungorum and include order, family, genus, and species (all belonging to phylum Ascomycota). Taxonomic levels of insect hosts were generally based on the GBIF Taxonomic Backbone and include order, family, genus, and species. In addition, ant hosts (Formicidae) were provided an additional three levels based on the taxon home pages of AntWiki^44^: subfamily, tribe, and subtribe. Index Fungorum, GBIF, and AntWiki represent leading data sources for their respective areas and are maintained continuously.

Countries of observations were updated according to currently recognized full UN member states. For biogeographical reasons, some regions/islands were specified if their boundaries are confined in a single country. If the location concerns a region that is not confined within a single country, it was placed between quotation marks (e.g., “New Guinea”, being an island bearing multiple national boundaries). Our database operates on a country level, as geographic coordinates were absent for the majority of observations.

Each record in EntomoFun 1.0 represents a unique combination of information variables based on the reported taxon names to maximize information. Multiple records of the same source type with identical information were merged in a single record, with all source references listed. Multiple records of different source types with identical information underwent filtering by level of scientific importance, according to the following order of priority (from high priority to low priority): Journals & Books, Preprints, Herbaria, Theses, Reports, Botanical Gardens, Conference Proceedings. We assigned two different categories to these source types, to reflect scientific quality, “1” for Journals & Books, Preprints, and Herbaria; and “0” for Theses, Reports, Botanical Gardens, and Conference Proceedings. The reason behind this is that several of the theses, reports, lists from botanical gardens, and conference proceedings presented observations that were incorrect and needed manual corrections. Nested duplicate records, i.e., those that hold less information (on taxonomy, location, or host life stage), were removed from the database. Only the most complete record was retained.

Any records presenting unverified or questionable information were identified and separated from the database. However, these were retained in a separate file (Unverified_data_records_file_four). Examples are records with inconsistent information or records taken from secondary literature that had incorrectly copied data from the primary literature. This also includes host associations that are unverified by the source or resulting from artificial/non-natural context.

**Fig. 1:**
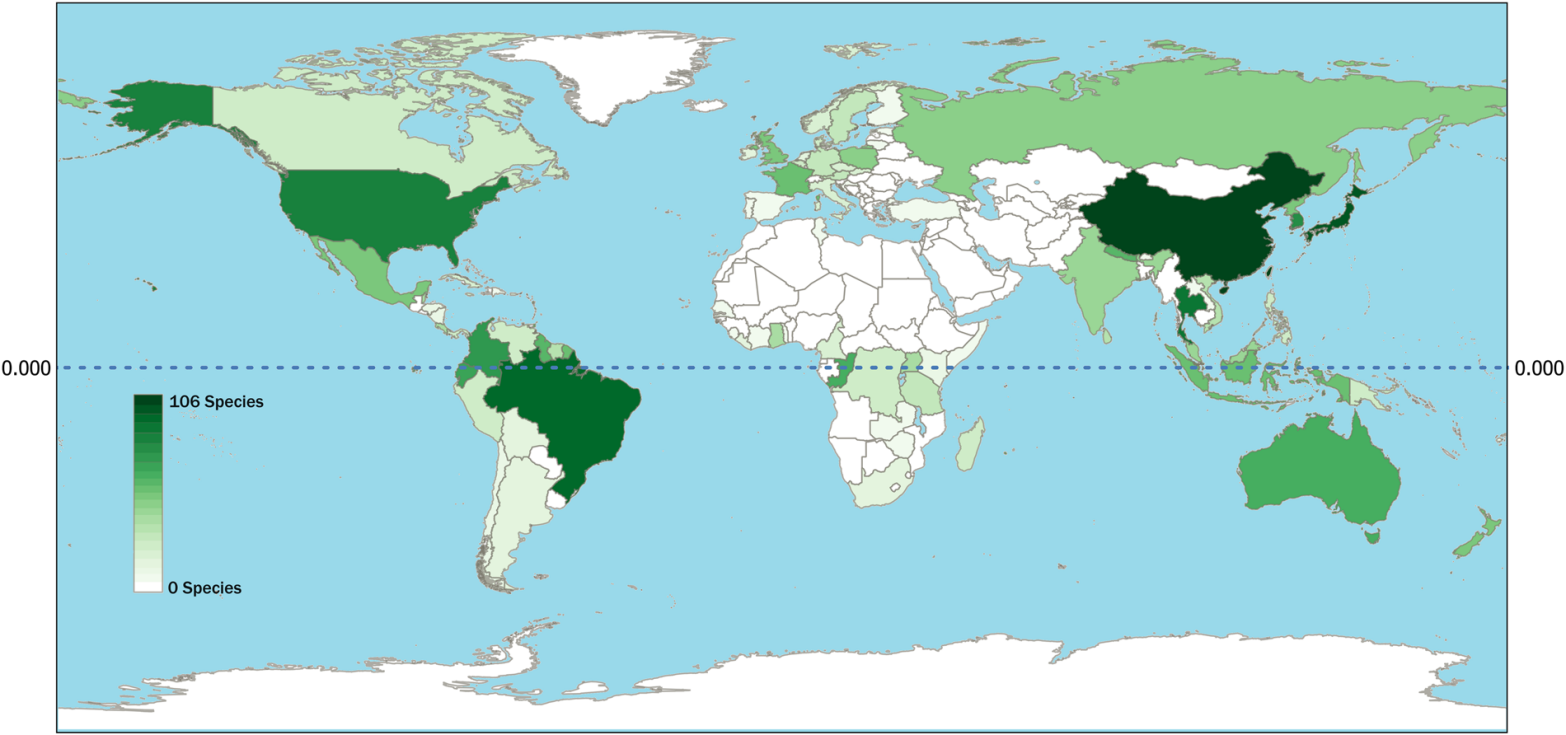
Global map showing the number of EF species recorded per country. National borders are according to currently recognised full UN member states. This map is based on 817 records. Only clear national boundaries are included; records not confined to a single country were excluded.

**Fig. 2:**
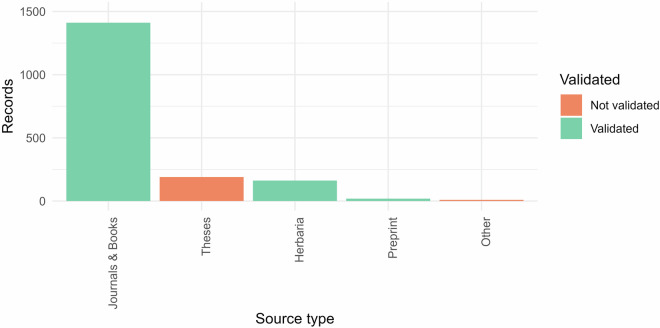
Origin of records per source type according to validation level. Colors correspond to Not validated “1” and Validated “0” source types. Source type “Other” includes the following three categories: Reports, Botanical garden checklists, and Conference proceedings.

**Fig. 3:**
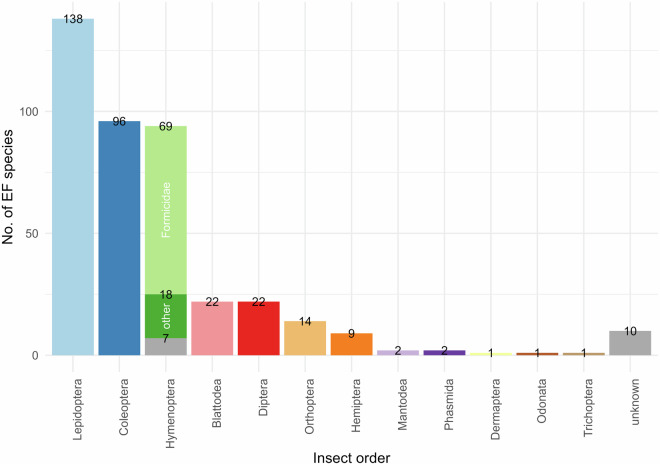
The number of EF species infecting each insect order. Hymenoptera hosts are split into Formicidae, other, and unknown (with respective counts).

**Fig. 4:**
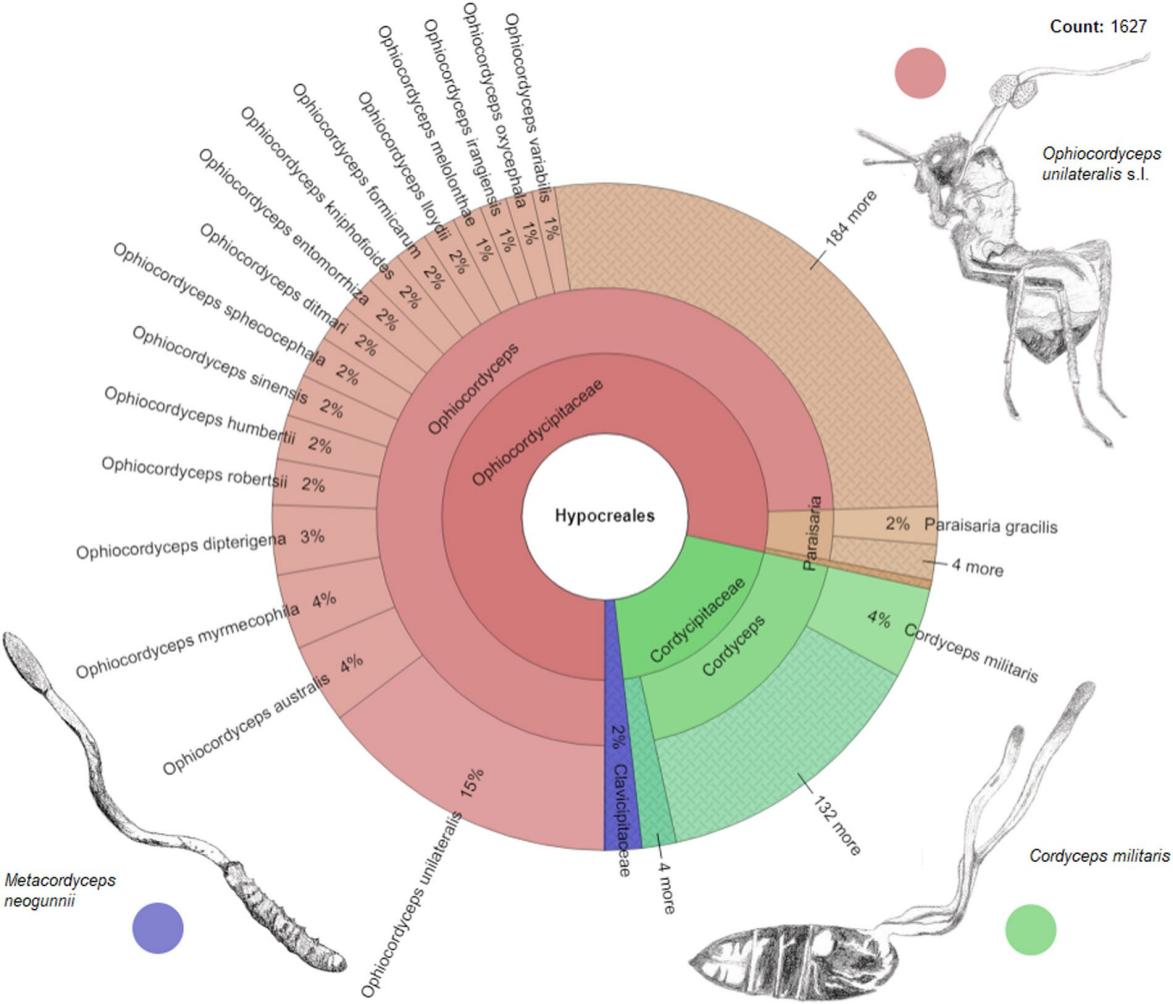
The proportional representation of records of EF species in the EntomoFun 1.0 database. Proportions are presented in this Krona chart. These are records with unique combinations of EF species, host taxon, country and host life stage. For illustrative purposes, any sensu lato species identities are not separated from the species level. Bands show levels of EF taxonomy, in the following order (inner to outer band): Order, Family, Genus, Species. Ophiocordycipitaceae (78%); Cordycipitaceae (20%); Clavicipitaceae (2%). Illustrations are provided per family, indicated by the respective color.

**Fig. 5:**
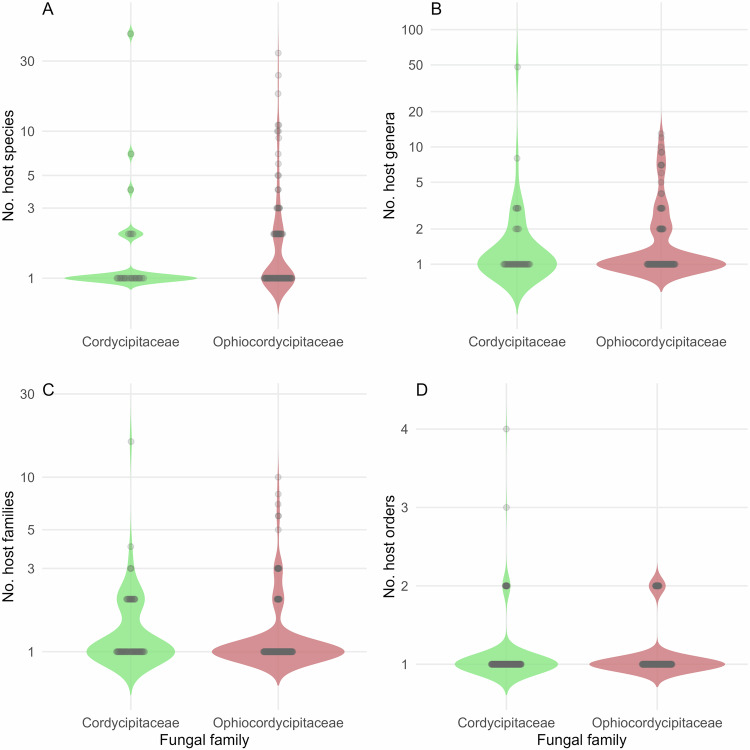
Host ranges of Cordicipitaceae and Ophiocordycipitaceae over multiple taxonomic levels. Distribution of EF species over the reported number of host taxa infected, categorized by fungal family. Based on validated records only (validation = 1). Lower taxon information was not available for all records, which means that e.g. the range of species number in image A is lower than the range of genera number in image B. Note that the y-axes of graphs A, B and C have a logarithmic scale. (**A**) Number of insect species infected by each EF species by fungal family (121 EF species out of a total of 371 species had species level host records; 33%). (**B**) Number of insect genera infected by each EF species by fungal family (147 of total 371 EF species = 40%). (**C**) Number of insect families infected by each EF species by fungal family (220 of total 371 EF species = 60%). (**D**) Number of insect orders infected by each EF species by fungal family (343 of total 371 EF species = 93%).

**Fig. 6:**
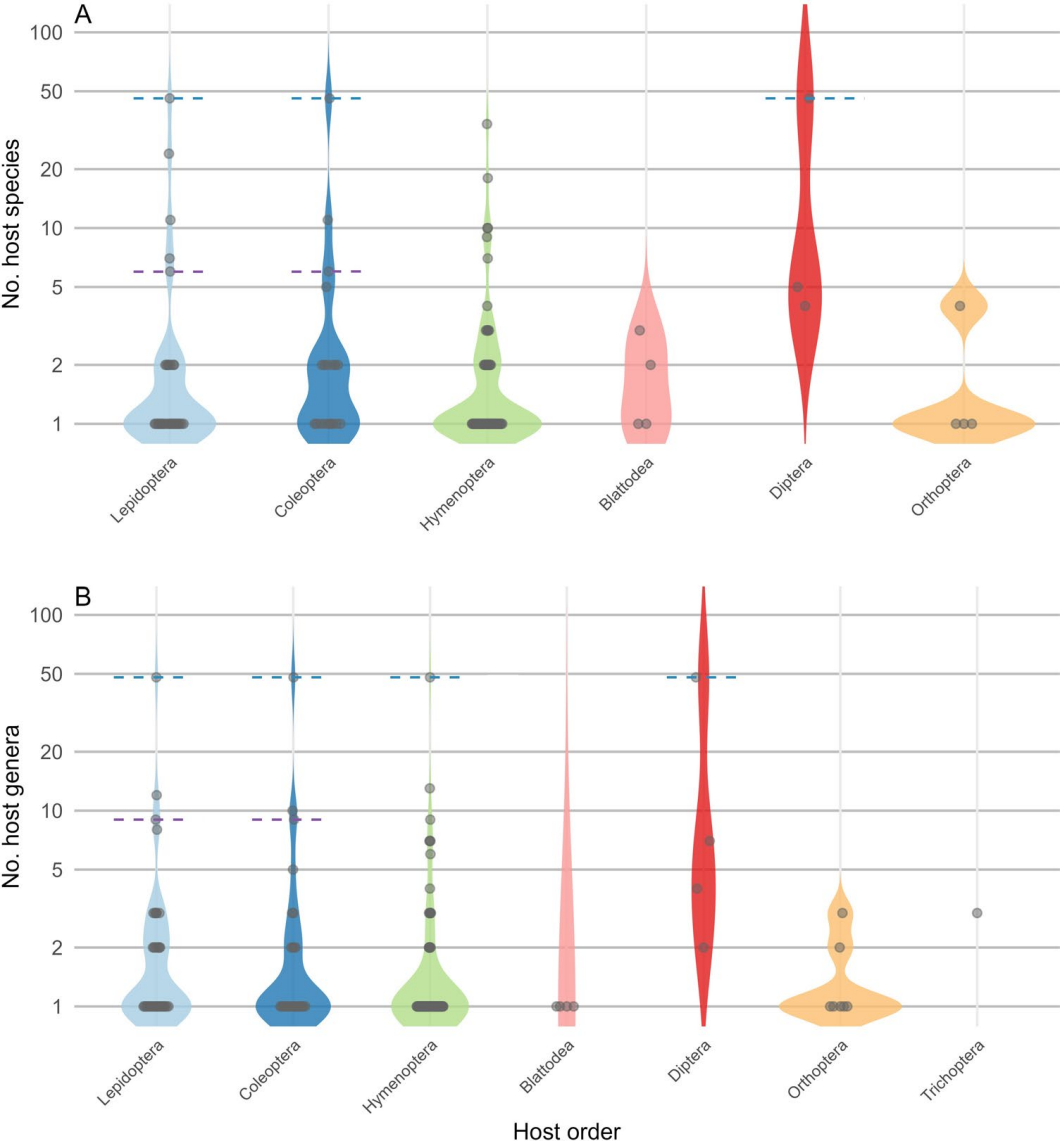
Insect orders and host range of EF species attacking them. Each point represents an EF species, and the total number of host species (**A**) or genera (**B**) shown across insect orders. Based on validated (1) records only. Hemiptera are not included here, as they were not thoroughly investigated. To enhance interpretation, the dashed lines indicate single generalist EF species with high host ranges across multiple host orders: *Cordyceps militaris* (blue) and *Paraisaria gracilis* (purple).

**Fig. 7:**
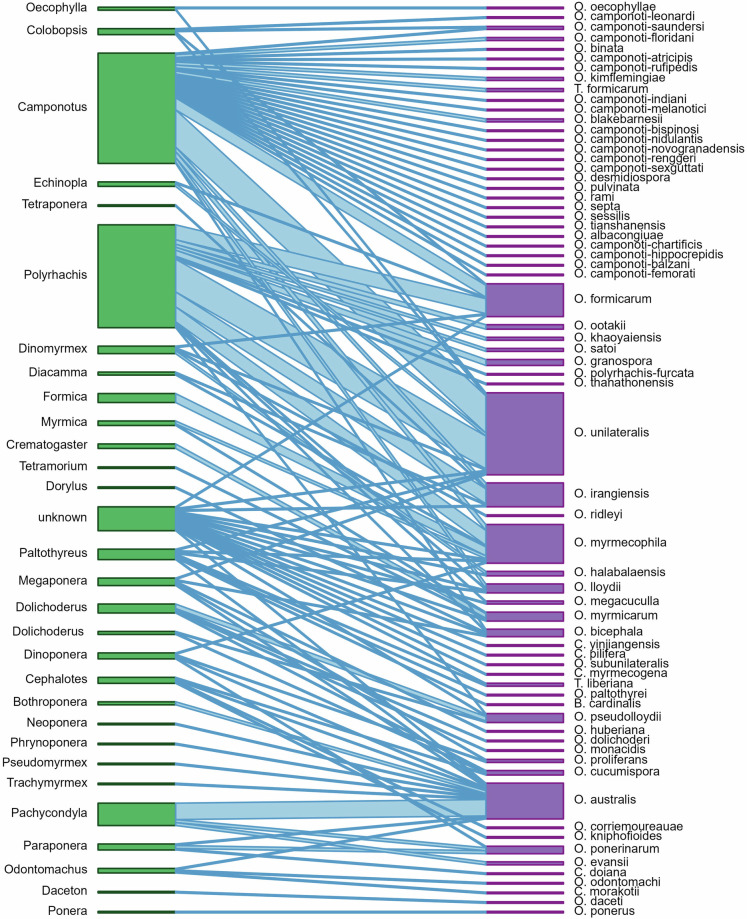
Bipartite network displaying reports of ant species infected by respective EF species, per ant genus. The width of a connection is proportional to the number of ant species in that genus infected by a fungal species.

To facilitate use of EntomoFun 1.0, we visualized some of the contents of the refined database (file_one). Only records with full taxon identities were considered for the figures (excluding “s.l.”, “cf.”, or “/” records), unless specified otherwise in the caption. Figures were made using R language and environment for statistical computing^45^, unless stated otherwise below. The global distribution of known EF species richness in the order Hypocreales is presented per country (Fig. 1). This was done in QGIS 3.28.9, using the default global basemap and by synchronizing the default countries with the currently accepted UN standards. The origin of records in the database is shown per source type and according to validation score (“1” or “0”; Fig. 2). The number of EF species infecting each insect order is presented as a bar plot (Fig. 3). A Krona chart^46^ shows the proportional representation of records of EF species in the database (Fig. 4). The violin plots depict the number of host species, genera, families, and orders for the EF families Cordicipitaceae and Ophiocordycipitaceae (Fig. 5) and the number of host species and genera attacked by EF species for each insect order (Fig. 6). The bipartite plot shows EF species with their ant genera (Fig. 7).

## Data Records

The EntomoFun 1.0 database^29^ is stored in Dryad as six different files (10.5061/dryad.1zcrjdg17). File one concerns the full database and presents all validated records. File two presents summaries of hosts infected per EF species. Similarly, a third file shows summaries of EF species affecting per host species. Note that the information presented in files 2 and 3 is also available from file 1; we present the data in these alternative formats for user convenience. File four presents records that are deemed doubtful, providing details for each to support this classification. File five presents a raw version of the database before removal of duplicate records, but is identical in all other respects to the first file. File six presents a list of all EF species currently described, listing the basionym and synonyms (based on sexual morphs only and expanded from the supplementary data by Araújo and Hughes^9^). All files are in .xlsx format. During the process of data collation, 1,100+ literature sources were examined, from which 600+ present observations that are included in the database. EntomoFun 1.0 currently has 1,791 records in file one, and an additional 139 unverified records (in file_four).

### File one: Refined database

The complete database collated during this study. In total, 1,791 records are presented for 371 EF species. The source type should be considered when using the data, although none of the records in file one show signs of being doubtful.

- Source_Type: Type of source presenting the record. Categories: Journals & Books, Herbaria, Preprints, Theses, Reports, Botanical Gardens, and Conference Proceedings.
- Validated: Indicating whether the record originates from a scientifically trustworthy source type. Categories: “1” for Journals & Books, Herbaria, and Preprints; “0” for Theses, Reports, Botanical Gardens, and Conference Proceedings.
- Source: Shortened citation of the primary source. Multiple sources are listed for multiple observations with identical information.
- Family_F: Fungal family. Levels: Clavicipitaceae, Cordycipitaceae, and Ophiocordycipitaceae
- Genus_F: Fungal genus. E.g., *Cordyceps*, *Ophiocordyceps*, *Torrubiella*.
- Species_F: Fungal species. E.g., *Cordyceps brasiliensis*, *Ophiocordyceps unilateralis*.
- Class_H: Host class.
- Order_H: Host order.
- Family_H: Host family.
- Subfamily_H: Host subfamily.
- Tribe_H: Host tribe.
- Subtribe_H: Host subtribe (in AntWiki^44^ referred to as alliance).
- Genus_H: Host genus.
- Species_H: Host species.
- Location: Location of observation on a national level.
- Life_Stage_H: Life stage of the infected host: Egg, larva, nymph, pupa, adult. Uncertainty indicated using multiple categories where appropriate.
- Reported_F: Fungal name reported by primary source.
- Reported_H: Host name reported by primary source.
- Level_Reported_H: Taxonomic level of reported host. This is important as some taxonomic levels are not considered by the GBIF Backbone Taxonomy^37^ (e.g., subfamily).
- Level_H: Taxonomic level of the host level included in the GBIF Backbone Taxonomy.

### File two: Summary of hosts per fungal species

A summary of the hosts affected by each recorded fungal species, added as a separate file to facilitate interpretation and consultation of the database.

- Species_F: Fungal species.
- Order_H: List of infected host orders.
- Species_H: List of infected host species. An asterisk (*) indicates hosts reported in source types for which “Validated” = 0.

### File three: Summary of fungal species per host species

A list summarizing the fungal species attacking each host species, added as a separate file to facilitate interpretation and consultation of the database.

- Species_H: Host species.
- Family_F_count: Count of fungal families attacking each host species.
- Species_F: List of fungal species attacking each host species.

### File four: Unverified records

A list of unverified and questionable records obtained during data collation. The same structure of file one is used, with some changes: the columns ‘Source_Type’ and ‘Validated’ are removed; two additional columns ‘Reasoning’ and ‘Information’ are included.

- Reasoning: The reason behind exclusion of each record from the refined database. Categories: “Doubtful record”, “EF species/variant non-existent”, “Host species non-existent”, “No observation/assumption”, “Lab/artificial”, “Uncertain record”.
- Information: Elaborate explanation for assigning doubtful status to respective records.

### File five: Raw database

Unrefined version of file one, in which unverified and nested duplicate records are not removed. This raw database thus retains additional records that present less specific information than is already available in records for EF–host associations per country and host life stage.

- Source_Type: Type of source presenting the record. Categories: Journals & Books, Herbaria, Preprints, Theses, Reports, Botanical Gardens, and Conference Proceedings.
- Validated: Indicating whether the record originates from a scientifically trustworthy source type. Categories: “1” for Journals & Books, Herbaria, and Preprints; “0” for Theses, Reports, Botanical Gardens, and Conference Proceedings.
- Reasoning: The reason behind exclusion of each record from the refined database. Categories: “Doubtful record”, “EF species/variant non-existent”, “Host species non-existent”, “No observation/assumption”, “Lab/artificial”, “Uncertain record”.
- Information: Elaborate explanation for assigning doubtful status to respective records.
- Source: Shortened citation of the primary source. Multiple sources are listed for multiple observations with identical information.
- Family_F: Fungal family. Levels: Clavicipitaceae, Cordycipitaceae, and Ophiocordycipitaceae
- Genus_F: Fungal genus.
- Species_F: Fungal species.
- Class_H: Host class.
- Order_H: Host order.
- Family_H: Host family.
- Subfamily_H: Host subfamily.
- Tribe_H: Host tribe.
- Subtribe_H: Host subtribe (in AntWiki^44^ referred to as alliance).
- Genus_H: Host genus.
- Species_H: Host species.
- Location: Location of observation on a national level.
- Life_Stage_H: Life stage of the infected host: Egg, larva, nymph, pupa, adult. Uncertainty indicated using multiple categories where appropriate.
- Reported_F: Fungal name reported by primary source.
- Reported_H: Host name reported by primary source.
- Level_Reported_H: Taxonomic level of reported host. This is important as some taxonomic levels are not considered by the GBIF Backbone Taxonomy^37^ (e.g., subfamily).
- Level_H: Taxonomic level of the host level included in the GBIF Backbone Taxonomy.

### File six: Expanded list of Hypocreales EF species

A list of all EF species that are currently described in Hypocreales (except Polycephalomycetaceae). Each EF species is presented with its basionym and synonyms, based on sexual morphs only and expanded from the supplementary data by Araújo and Hughes^9^.

- Species_Araujo_and_Hughes_2016: Species name as in Araújo and Hughes^9^ before expansion. “NA” value = species added in our database that was not present in Araújo and Hughes^9^.
- Current_Name: Displays the currently accepted species name of the fungus.
- Basionym: Displays the basionym of each species.
- Synonyms: Lists synonyms of each species.
- Protologue: Reference in which the species was formally described (validly published).
- Additional_Reference: Additional literature referring to the species.

## Technical Validation

The EntomoFun 1.0 database largely consists of records obtained from the literature. Data records went through rigorous quality control. Firstly, we checked if the originally reported name corresponded with taxon nomenclature of the respective publication year (with Index Fungorum^34^) for all records. Any inconsistencies were resolved. Secondly, the information content was double-checked for over half of all records by returning to the primary literature. This additional step was performed to validate our methodology.

Data quality can be distinguished by using the “Validated” variable. Our database thus provides a complete repository of observations while allowing filtering based on information quality. Unverified and questionable records were separated and are not included in the final version of the refined database. In other words, there are actually three levels of quality for records in our database: validated (“1”), unvalidated (“0”) (both included in file one), and unverified (included in file four).

Data were collected from the literature according to a standardized methodology (Figs. 1–2). The collation of data included any historical synonyms of EF species, disregarding whether or not this would result in additional records. These were later updated according to current accepted nomenclature, and any resulting duplicates were removed (these are still included in file five). By using a broader scope on nomenclature, extra records were obtained that would otherwise have been overlooked. Multiple other steps described in the methods section further contributed to a virtually complete repository of published records on EF species of Hypocreales.

## Usage Notes

All files included in EntomoFun 1.0 are publicly available in Dryad under a Creative Commons Zero (CC0) license (https://creativecommons.org/public-domain/cc0/). This database adheres to the FAIR principles^27^, allowing researchers to find, access, understand, and reuse data related to EF. Authors may freely use our database with the condition that this paper is cited.

Standardized data collation was employed from the literature. EntomoFun 1.0 contains virtually every known association between teleomorph EF species in Hypocreales (excluding Polycephalomycetaceae) and their respective host taxa, as well as the host life stage and geographical distribution. Having this information centralized provides a significant advantage when studying EF ecology, host range, and biogeography (as explained in the Summary and Background section). Our database can help identify knowledge gaps to direct future studies. For example, the distributional gaps identified by our data (i.e., Wallacean shortfall^24^; Fig. 1) highlights regions where additional samples are needed for future EF studies. When using EntomoFun 1.0 for ecological analyses, users are advised to take into consideration these knowledge gaps.

Because of cryptic diversity, some EF species have been – or might be in the future – split into multiple species. Depending on the use of this database, “older” species reports might be considered as sensu lato. Notable examples are: *Ophiocordyceps australis*, *O. dipterigena*, *O. myrmecophila*, *O. sphecocephala*, and *O. unilateralis*. This is a consideration that should also be made for host taxa, e.g., for ants (Formicidae) of the genera *Camponotus* and *Pachycondyla*.

## Data Availability

No custom code was used to create the presented database.

## References

[1] Singh, B. K. *et al*. Climate change impacts on plant pathogens, food security and paths forward. *Nat. Rev. Microbiol.* **21**, 640–656 (2023). 37131070 10.1038/s41579-023-00900-7 PMC10153038

[2] Lyles, A. M. & Dobson, A. P. Infectious Disease and Intensive Management: Population Dynamics, Threatened Hosts, and Their Parasites. *J. Zoo Wildl. Med.* **24**, 315–326 (1993).

[3] Woolhouse, M. E. J., Taylor, L. H. & Haydon, D. T. Population Biology of Multihost Pathogens. *Science* **292**, 1109–1112 (2001). 11352066 10.1126/science.1059026

[4] Dwyer, G. Density Dependence and Spatial Structure in the Dynamics of Insect Pathogens. *Am. Nat.* **143**, 533–562 (1994).

[5] Liu, W.-C., Bonsall, M. B. & Godfray, H. C. J. The form of host density-dependence and the likelihood of host–pathogen cycles in forest-insect systems. *Theor. Popul. Biol.* **72**, 86–95 (2007). 17298839 10.1016/j.tpb.2007.01.002

[6] Greer, A. L., Briggs, C. J. & Collins, J. P. Testing a key assumption of host‐pathogen theory: density and disease transmission. *Oikos* **117**, 1667–1673 (2008).

[7] Burdon, J. J. & Thrall, P. H. Pathogen evolution across the agro‐ecological interface: implications for disease management. *Evol. Appl.* **1**, 57–65 (2008). 25567491 10.1111/j.1752-4571.2007.00005.x PMC3352394

[8] Hortal, J. *et al*. Seven Shortfalls that Beset Large-Scale Knowledge of Biodiversity. *Annu. Rev. Ecol. Evol. Syst.* **46**, 523–549 (2015).

[9] Araújo, J. P. M. & Hughes, D. P. Diversity of Entomopathogenic Fungi: Which Groups Conquered the Insect Body? in *Advances in Genetics* vol. 94 1–39 (Elsevier, 2016). 10.1016/bs.adgen.2016.01.001 27131321

[10] Kaishian, P. *et al*. Definitions of parasites and pathogens through time. Preprint at 10.22541/au.165712662.22738369/v2 (2024).

[11] Mora, M. A. E., Castilho, A. M. C. & Fraga, M. E. Classification and infection mechanism of entomopathogenic fungi. *Arq. Inst. Biológico* **84** (2018).

[12] Andersen, S. B. *et al*. The Life of a Dead Ant: The Expression of an Adaptive Extended Phenotype. *Am. Nat.* **174**, 424–433 (2009). 19627240 10.1086/603640

[13] De Bekker, C., Beckerson, W. C. & Elya, C. Mechanisms behind the Madness: How Do Zombie-Making Fungal Entomopathogens Affect Host Behavior To Increase Transmission? *mBio* **12**, e01872–21 (2021). 34607463 10.1128/mBio.01872-21 PMC8546595

[14] Rosenberg, Y. *et al*. The global biomass and number of terrestrial arthropods. *Sci. Adv.* **9**, eabq4049 (2023). 36735788 10.1126/sciadv.abq4049 PMC9897674

[15] Stork, N. E. How Many Species of Insects and Other Terrestrial Arthropods Are There on Earth? *Annu. Rev. Entomol.* **63**, 31–45 (2018). 28938083 10.1146/annurev-ento-020117-043348

[16] St. Leger, R. J. Insects and their pathogens in a changing climate. *J. Invertebr. Pathol.* **184**, 107644 (2021). 34237297 10.1016/j.jip.2021.107644

[17] Fournier, A., Penone, C., Pennino, M. G. & Courchamp, F. Predicting future invaders and future invasions. *Proc. Natl. Acad. Sci.* **116**, 7905–7910 (2019). 30926662 10.1073/pnas.1803456116 PMC6475384

[18] Timms, R. & Read, A. F. What makes a specialist special? *Trends Ecol. Evol.* **14**, 333–334 (1999). 10441304 10.1016/s0169-5347(99)01697-3

[19] Meyling, N. V. & Hajek, A. E. Principles from community and metapopulation ecology: application to fungal entomopathogens. *BioControl* **55**, 39–54 (2010).

[20] Tylianakis, J. M., Laliberté, E., Nielsen, A. & Bascompte, J. Conservation of species interaction networks. *Biol. Conserv.* **143**, 2270–2279 (2010).

[21] Hajek, A. E., Junior, I. D. & Butler, L. Entomopathogenic Fungi as Classical Biological Control Agents. in *Environmental Impacts of Microbial Insecticides* (eds. Hokkanen, H. M. T. & Hajek, A. E.) 15–34, 10.1007/978-94-017-1441-9_2 (Springer Netherlands, Dordrecht, 2003).

[22] Evans, H. C. Entomogenous fungi in tropical forest ecosystems: an appraisal. *Ecol. Entomol.* **7**, 47–60 (1982).

[23] Quandt, C. A. & Haelewaters, D. Phylogenetic Advances in Leotiomycetes, an Understudied Clade of Taxonomically and Ecologically Diverse Fungi. in *Encyclopedia of Mycology* 284–294, 10.1016/B978-0-12-819990-9.00052-4 (Elsevier, 2021).

[24] Haelewaters, D. *et al*. Biological knowledge shortfalls impede conservation efforts in poorly studied taxa—A case study of Laboulbeniomycetes. *J. Biogeogr.* **51**, 29–39 (2024).

[25] Reichman, O. J., Jones, M. B. & Schildhauer, M. P. Challenges and Opportunities of Open Data in Ecology. *Science* **331**, 703–705 (2011). 21311007 10.1126/science.1197962

[26] Dorey, J. B. *et al*. A globally synthesised and flagged bee occurrence dataset and cleaning workflow. *Sci. Data* **10**, 747 (2023). 37919303 10.1038/s41597-023-02626-w PMC10622554

[27] Wilkinson, M. D. *et al*. The FAIR Guiding Principles for scientific data management and stewardship. *Sci. Data* **3**, 160018 (2016). 26978244 10.1038/sdata.2016.18 PMC4792175

[28] *International Code of Nomenclature for Algae, Fungi, and Plants*. vol. 159 (Koeltz Botanical Books, 2018).

[29] De Wint, F. C. *et al*. EntomoFun 1.0: A global database of entomopathogenic fungi and associations with their hosts. 513216 bytes *Dryad*10.5061/DRYAD.1ZCRJDG17 (2024).

[30] Xiao, Y.-P. *et al*. Polycephalomycetaceae, a new family of clavicipitoid fungi segregates from Ophiocordycipitaceae. *Fungal Divers.* **120**, 1–76 (2023).

[31] Cannon, P. F. & Kirk, P. M. The philosophy and practicalities of amalgamating anamorph and teleomorph concepts. *Stud. Mycol*. 19–26 (2000).

[32] Hawksworth, D. L. *et al*. The Amsterdam Declaration on Fungal Nomenclature. *IMA Fungus* **2**, 105–111 (2011). 22679594 10.5598/imafungus.2011.02.01.14 PMC3317370

[33] Robert, V. *et al*. MycoBank gearing up for new horizons. *IMA Fungus* **4**, 371–379 (2013). 24563843 10.5598/imafungus.2013.04.02.16 PMC3905949

[34] Index Fungorum https://www.indexfungorum.org/Names/Names.asp (2024).

[35] Sharkey, M. J. Phylogeny and Classification of Hymenoptera*. *Zootaxa* **1668** (2007).

[36] Chamberlain, S. *et al*. R package ‘taxize’ (2020).

[37] Registry-Migration.Gbif.Org. GBIF Backbone Taxonomy. GBIF Secretariat 10.15468/39OMEI (2023).

[38] Schoch, C. L. *et al*. NCBI Taxonomy: a comprehensive update on curation, resources and tools. *Database* **2020**, baaa062 (2020). 32761142 10.1093/database/baaa062 PMC7408187

[39] Sung, G.-H. *et al*. Phylogenetic classification of Cordyceps and the clavicipitaceous fungi. *Stud. Mycol.* **57**, 5–59 (2007). 18490993 10.3114/sim.2007.57.01 PMC2104736

[40] Kobayasi, Y. The genus Cordyceps and its allies. *Sci. Rep. Tokyo Bunrika Daigaku* **Section B**, 53–260 (1941).

[41] Rose, E. A. F., Harris, R. J. & Glare, T. R. Possible pathogens of social wasps (Hymenoptera: Vespidae) and their potential as biological control agents. *N. Z. J. Zool.* **26**, 179–190 (1999).

[42] Hywel-Jones, N. Cordyceps sphecocephala and a Hymenostilbe sp. infecting wasps. *Mycol. Res*. 154–158 (1995).

[43] Petch, T. Notes on entomogenous fugi. *Trans. Br. Mycol. Soc*. 49–75 (1933).

[44] Antwiki http://Antwiki.org (2024).

[45] R Core Team. R: A language and environment for statistical computing. R Foundation for Statistical Computing (2022).

[46] Ondov, B. D., Bergman, N. H. & Phillippy, A. M. Interactive metagenomic visualization in a Web browser. *BMC Bioinformatics* **12** (2011). 10.1186/1471-2105-12-385 PMC3190407 21961884

